# Luminophores in the fur of seven Australian Wet Tropics mammals

**DOI:** 10.1371/journal.pone.0320432

**Published:** 2025-04-30

**Authors:** Linda M. Reinhold, Tasmin L. Rymer, David T. Wilson

**Affiliations:** 1 College of Science and Engineering, James Cook University, Cairns, Queensland, Australia; 2 College of Public Health, Medical and Veterinary Sciences, James Cook University, Cairns, Queensland, Australia; 3 Centre for Tropical Environmental and Sustainability Sciences, James Cook University, Cairns, Queensland, Australia; 4 Centre for Molecular Therapeutics, Australian Institute of Tropical Health and Medicine, James Cook University, Cairns, Queensland, Australia; Konkuk University - Seoul Campus: Konkuk University, REPUBLIC OF KOREA

## Abstract

Bright photoluminescence in the fur of mammals has recently raised considerable scientific interest. The fur of many mammal species, including Australian northern long-nosed (*Perameles pallescens*) and northern brown (*Isoodon macrourus*) bandicoots, photoluminesces strongly, displaying pink, yellow, blue and/or white colours. We used reversed-phase high-performance liquid chromatography and electrospray ionisation mass spectrometry to investigate the luminophores contributing to this photoluminescence. At least two classes of luminophore were observed in bandicoot fur extracts, and four of the orange-pink photoluminescent molecules had molecular masses consistent with protoporphyrin, coproporphyrin, uroporphyrin and heptacarboxylporphyrin isomers. Fur extracts of three other species of marsupial, a placental and a monotreme also contained a luminophore consistent with the molecular mass of protoporphyrin, whether or not pink photoluminescence was evident in the pelage as a whole. This study is the first chemical analysis of luminophores contributing to photoluminescence in the fur of Australasian mammals since two tryptophan metabolites were identified more than 50 years ago.

## Introduction

Photoluminescence (fluorescence and/or phosphorescence) is the re-emission of absorbed photons from matter, usually at a higher wavelength than the absorption wavelength [1]. It occurs in many animals (e.g., fish [2–4], amphibians [5–7], reptiles [8–10] birds [11–13]), including mammals [14–16]. The photoluminescence of mammals has been documented worldwide and is a common characteristic of fur and other biological tissues, both externally and internally [5,17]. While the hair chemistry of humans and production fur animals has been extensively analysed [18–20], particularly for sheep’s (*Ovis aries*) wool [21], the fur composition of other animals has not been examined as thoroughly.

**Fig. 1:**
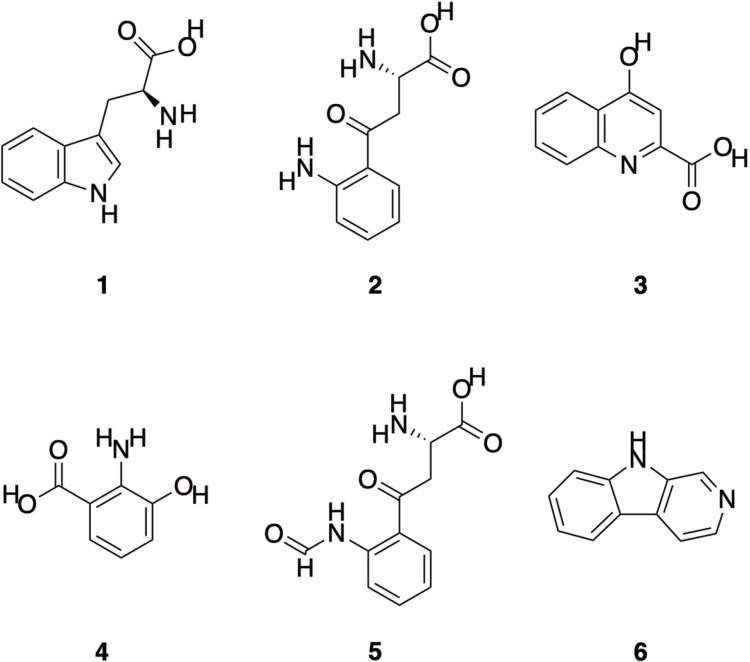
Molecular structure of tryptophan and some tryptophan metabolites identified in mammalian pelage. Tryptophan (1; chemical formula =  C_11_H_12_N_2_O_2_; exact mass =  204.0899 Da). Kynurenine (2; C_10_H_12_N_2_O_3_; 208.0848 Da). Kynurenic acid (3; C_10_H_7_NO_3_; 189.0426 Da). 3-Hydroxyanthranilic acid (4; C_7_H_7_NO_3_; 153.0426 Da). *N*-formylkynurenine (5; C_11_H_12_N_2_O_4_; 236.0797 Da). Beta-carboline (6; C_11_H_8_N_2_; 168.0687 Da).

The colouration of fur in white light is typically a consequence of eumelanin (black or brown pigmentation), pheomelanin (red and yellow colouration) [22], trichosiderin [23,24] or cinnabarinic acid (contributes to red colouration) [25]. However, Bolliger (1944) noted that otherwise colourless components, termed ‘fluorescent compounds’, contributed to bright photoluminescence when incorporated into the fur structure of some mammals [26]. Work on identifying such luminophores in mammal fur began in the 1950s [27], with studies showing that the photoluminescence of laboratory rats (*Rattus norvegicus*) was predominantly caused by the tryptophan (**1**) metabolite kynurenine (**2**) (Fig. 1) and, to a lesser extent, by the tryptophan metabolites kynurenic acid (**3**) and *N*-acetylkynurenine [28,29].

Kynurenine (**2**), along with 3-hydroxyanthranilic acid (**4**) (Fig. 1), was later extracted from the blue-photoluminescent fur of New Guinean Goodfellow’s tree-kangaroos (*Dendrolagus goodfellowi*) [25]. 3-Hydroxyanthranilic acid (**4**) was also extracted from the blue-photoluminescent fur of Australian common brushtail possums (*Trichosurus vulpecula*), although the full suite of luminophores present was not identified [25]. Pine et al. (1985) also found 3-hydroxyanthranilic acid (**4**) in the blue-photoluminescent fur of South American bare-tailed woolly opossums (*Caluromys philander*) [14]. Additional tryptophan metabolites, *N*-formylkynurenine (**5**) and β-carboline 1,3-dicarboxylic acids (**6**) (Fig. 1), were isolated from blue- and/or cyan-photoluminescent sheep’s wool [30,31], but even for this most-studied species, identification of the full complement of luminophores remains uncertain [32,33]. The photoluminescent colour of a tryptophan metabolite luminophore extracted in solution does not necessarily indicate the photoluminescent colour of the fur *in situ*, and in some cases the same tryptophan metabolite may photoluminesce in different colours, dependent on its microenvironment [30].

**Fig. 2:**
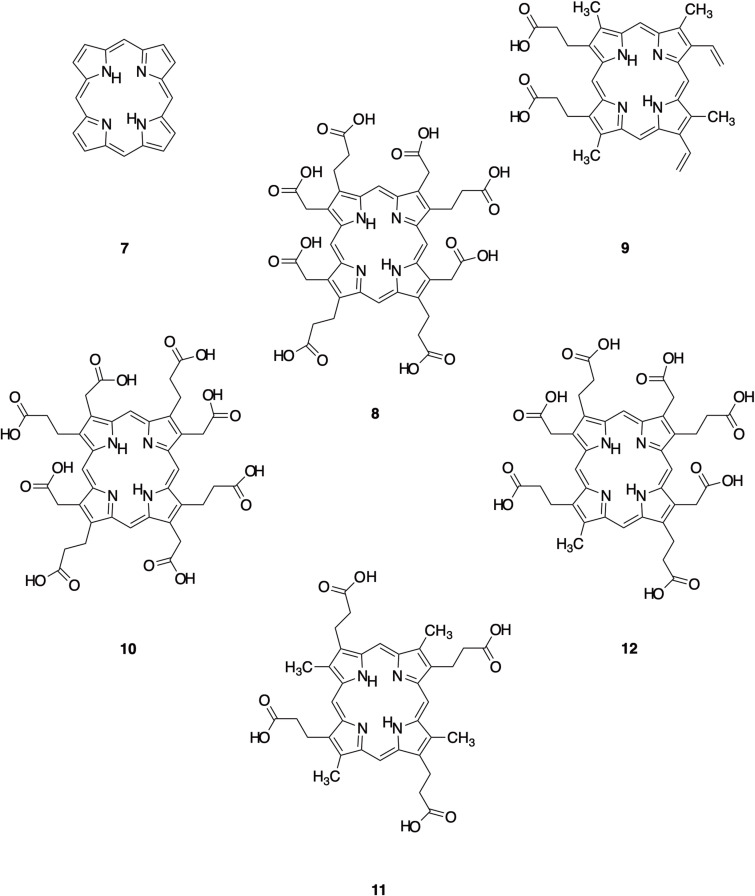
Molecular structure of porphin and some porphin derivatives identified in mammalian pelage. Porphin (7; chemical formula =  C_20_H_14_N_4_; exact mass =  310.1218 Da). Uroporphyrin III (8; C_40_H_38_N_4_O_16_; 830.2283 Da). Protoporphyrin IX (9; C_34_H_34_N_4_O_4_; 562.2580 Da). Uroporphyrin I (10; C_40_H_38_N_4_O_16_; 830.2283 Da). Coproporphyrin I (11; C_36_H_38_N_4_O_8_; 654.2690 Da). Heptacarboxylporphyrin I (12; C_39_H_38_N_4_O_14_; 786.2385 Da).

Red-photoluminescent emission from a peak excitation wavelength of ~  400 nm and longer is the spectrographic signature of the porphyrin (substituted porphins (**7**)) class of luminophores (Fig. 2) [34–36]. The presence of porphyrins in mammal pelage was first suspected due to a red photoluminescence in the spines of European hedgehogs (*Erinaceus europaeus*) [11]. The luminophores were later confirmed by chemical analysis as coproporphyrin III, uroporphyrin III (**8**) and protoporphyrin IX (**9**) (Fig. 2, [35]). Spectroscopically, only protoporphyrin IX (**9**) was detected from this species [36]. Uroporphyrin I (**10**) or III (**8**) was detected by emission and excitation spectroscopy in Guyanan short-tailed opossums (*Monodelphis brevicaudata*) and Linnaeus’ mouse opossums (*Marmosa murina*, [36]). In addition, porphyrin S-411 (an analogue of coproporphyrin) was identified by emission and excitation spectroscopy in southern (*Glaucomys volans*) and northern (*G. sabrinus*) flying squirrel fur [36]. Coproporphyrin I (**11**), uroporphyrin I (**10**), uroporphyrin III (**8**) and heptacarboxylporphyrin (**12**) were identified by chemical analysis of the photoluminescent orange-red fur of a South African springhare (*Pedetes capensis*, [16]).

The fur of these porphyrin-photoluminescing species did not yield tryptophan metabolites, but non-porphyrin luminophores were not necessarily tested for. For example, the blueish fur photoluminescence of South African springhares appeared to only emit from museum specimens used in spectroscopy experiments, and not from captive animals from which a fur sample was taken for chemical analysis [16]. Additionally, in European hedgehog spines, the broad blue–cyan emission peak has been attributed to either keratins [36] or brown eumelanins [35], but these were not specifically identified.

Recent studies have used emission and excitation fluorescence spectrophotometry, spectroscopy, and spectrofluorimetry to match absorption and emission maxima of fur luminophores to wavelengths of known molecules, but not all photoluminescent compounds have distinct, identifiable spectrographic signatures [18,20,36]. Other studies have used thin-layer chromatography (TLC), high-performance liquid chromatography (HPLC), electrospray ionisation mass spectrometry (ESI-MS) and microplate reader fluorescence analysis of extractions from fur to identify luminophores, with mixed success [16,25,35,37]. Interestingly, the only porphyrin molecule identified in northern and southern flying squirrel fur by excitation spectroscopy [36] is different from the potential porphyrin found in these species by fourier transform-ion cyclotron resonance mass spectrometry (FT-ICR MS, [37]). In general, much remains unknown about luminophores in the fur of mammals.

The aim of this study was to identify luminophores contributing to photoluminescence, particularly pink photoluminescence, in Australian mammal fur. We used reversed-phase HPLC and ESI-MS to observe and identify luminophores initially in two Australian marsupials from the Wet Tropics region of Far North Queensland. Fur samples of both northern long-nosed (*Perameles pallescens*) and northern brown (*Isoodon macrourus*) bandicoots were used, and we hypothesised that porphyrins were the cause of the bright pink photoluminescence (at 395 nm excitation) observed in these samples, as suggested by Toussaint et al. (2023) for long-nosed bandicoots [36]. In addition, in northern brown bandicoots, varying colours are displayed as the animal turns and different parts of the fur shaft are angled to the viewer [38], suggesting that multiple luminophores may be present.

To determine whether the luminophores present in bandicoot fur might be common across multiple species, we compared the results from the two bandicoot species to five other species of Wet Tropics mammals: two marsupials that photoluminesce pink, the northern quoll (*Dasyurus hallucatus*) and the coppery brushtail possum (*Trichosurus johnstonii*); one marsupial that only photoluminesces blue, the Lumholtz’s tree-kangaroo (*Dendrolagus lumholtzi*); one placental that photoluminesces blue, the pale field rat (*Rattus tunneyi*); and one monotreme that photoluminesces silvery grey to dull green and mild pink, the platypus (*Ornithorhynchus anatinus*). We hypothesised that we would not detect porphyrins in those species that did not display strong pink photoluminescence.

## Materials and Methods

### Fur collection

Fur from a northern brown and a northern long-nosed bandicoot (both young males) was sourced from fresh roadkill animals collected at night around the Yungaburra and Lake Eacham/Tarzali (17°24’28.25“S, 145°35’49.75”E; 17°15’59.47”S, 145°31’25.72”E) area of the Atherton Tablelands in Far North Queensland, Australia. Each carcass was examined with 310 nm (Tao Yuan), 365 nm (OLight) and 395–410 nm (Dulex) torches and photographed with a Panasonic Lumix TZ-80 camera with no filters or post-processing. Clean but unwashed fur was shaved (blade 0 electric shaver) from the dorsal, flank and ventral surfaces of each animal. The fur samples were separately wrapped in aluminium foil to prevent photobleaching [39] and then sealed in separate resealable plastic bags. Samples were stored at -18°C until analysis. Fur samples of other species were variously sourced, either as fresh roadkill (female coppery brushtail possum, 17°25’53.81”S, 145°29’19.41”E; and female pale field rat, 17°02’56.10”S, 145°47’57.19”E), or as frozen specimens located at the James Cook University Nguma-bada campus (previously collected by other researchers – male northern quoll, male Lumholtz’s tree-kangaroo and female platypus). Fur was collected under Queensland Department of Environment and Science Research permit number WA0034269, granted to LMR under the *Nature Conservation (Animals) Regulation 2020*.

### Luminophore extraction

Luminophores were extracted from fur samples using conditions determined in preliminary extraction tests over a range of solvent and temperature conditions in our laboratory to provide the most efficient luminophore extraction. For each extraction, unwashed flank fur was weighed out into capped 15 mL polypropylene tubes and 20% trifluoroacetic acid (TFA; Auspep)/water was added until the fur was covered with the solvent. The ratio of mass of fur to volume of solvent was not the same for each species as quantitative differences between species were not being calculated. The amount of fur used was chosen to maximise the chances of identifying luminophores from each species. The masses of fur, volumes of solvent and extract used for each species are listed in S1 Table. Tubes were kept wrapped in aluminium foil whenever possible.

The tubes of fur and solvent were then heated at 95.0°C in a heat block for one hour, during which they were agitated briefly four separate times. If not used immediately, tubes were then stored in darkness at 4°C until analysis. Tubes were spun at 4000 rpm for five minutes to pellet the fur, then observed at 310–410 nm in a dark room. Extract supernatant (S1 Table) was transferred to clean separate tubes and syringe filtered (25 mm, 0.22 μm PVDF filter; Sartorius) into new 15 mL tubes prior to further use.

### Reversed-phase high-performance liquid chromatography (RP-HPLC)

Filtered fur extract (S1 Table) was loaded onto a reversed-phase column (Aeris peptide XB-C18, 250 ×  10 mm, 5 μm, 100 Å; Phenomenex) and run on an Agilent 1260 Infinity HPLC system (Agilent Technologies). A gradient from 0–80% Buffer B in 120 minutes [Buffer A, 0.05% TFA (Auspep)/H_2_O; Buffer B, 90% acetonitrile (ACN; Sigma-Aldrich)/10% H_2_O/0.045% TFA (Auspep)] was run at a flow rate of 3 mL/min. The absorbance was monitored at wavelengths of 214, 280, 330, 365, 380, 395 and 400 nm. Monitored absorption wavelengths of 214 and 280 nm were chosen as they are standard wavelengths used for peptides and proteins [40]. Wavelengths of 330 and 365 nm were chosen because they had previously been identified as peak excitation wavelengths for tryptophan-metabolite photoluminescence in fur [20,25,31], and 395 and 400 nm for excitation of pink-red porphyrin derivatives [15,35]. Fractions were collected every 30 seconds into 2 mL 96-well deep-well plates. Tray illumination was set to ‘off’ during collection of fractions. Deep-well plates containing fractions were observed under wavelengths of 310–410 nm and wells that showed the brightest photoluminescence, different colours and/or significant RP-HPLC peaks were transferred into individual 2 mL microcentrifuge tubes for further analysis. The most strongly coloured fractions were selected and photographed for each species. Photographs were taken with a Panasonic Lumix TZ-80 camera without any filters or post-processing.

### Liquid chromatography/electrospray ionisation – mass spectrometry (LC/ESI-MS)

The RP-HPLC fractions that showed the brightest photoluminescence, different colours and/or significant absorbance peaks from each species (northern long-nosed bandicoot: n =  7 fractions; northern brown bandicoot: n =  7; northern quoll: n =  6; coppery brushtail possum: n =  7; Lumholtz’s tree-kangaroo: n =  9; pale field rat: n =  8; platypus: n =  8) were loaded (50 μL) onto a reversed-phase column (Aeris peptide XB-C18, 150 ×  2.1 mm, 3.6 µm, 100 Å; Phenomenex) and analysed on a Shimadzu Prominence HPLC system coupled to a Shimadzu LCMS2020 mass spectrometer. A 1% gradient from 0–80% LCMS Buffer B in 80 minutes [LCMS Buffer A, 0.1% formic acid (FA; Sigma-Aldrich)/H_2_O; LCMS Buffer B, 90% ACN (LCMS grade; Thermo Fisher Scientific)/10% H_2_O/0.09% FA (Sigma-Aldrich)] was run at a flow rate of 0.25 mL/min. The absorbance was monitored at 330 nm and 400 nm, to cover maximal excitation wavelengths of expected tryptophan metabolites and porphyrin derivatives. Mass spectra were collected in positive and negative ionisation mode over a scan range of *m/z* 130–2000 and *m/z* 200–2000, respectively, with a detector voltage of 1.35 kV, nebulising gas flow of 1.5 L/min, and drying gas flow of 3 L/min. Data were collected and analysed using Shimadzu LabSolutions v5.96 software. A protoporphyrin IX standard (5 µ L of ~  1 mg/mL; Sigma-Aldrich) in both methanol-d4 (Cambridge Isotope Laboratories) and dimethyl sulfoxide-d6 (Cambridge Isotope Laboratories) was run under the same conditions.

## Results

### Fur collection

**Fig. 3:**
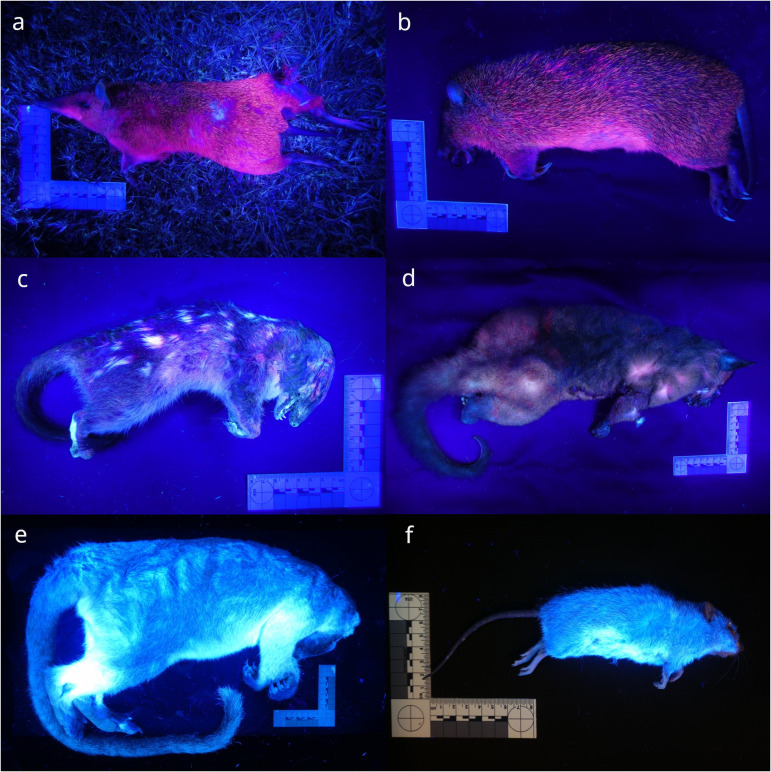
Marsupial and placental mammals used for fur luminophore extraction. Photoluminescence under ultraviolet-violet torchlight. (a) Fresh roadkill male northern long-nosed bandicoot (*Perameles pallescens*; 5 s exposure), on roadside grass. (b) Fresh roadkill male northern brown bandicoot (*Isoodon macrourus*; 6 s exposure). (c) Frozen-thawed male northern quoll (*Dasyurus hallucatus*; 10 s exposure). (d) Fresh roadkill female coppery brushtail possum (*Trichosurus johnstonii*; 10 s exposure). (e) Frozen-thawed male Lumholtz’s tree-kangaroo (*Dendrolagus lumholtzi*; 20 s exposure). (f) Fresh roadkill female pale field rat (*Rattus tunneyi*; 8 s exposure). (a)–(d) at 395–410 nm; (e)–(f) at 365 nm.

Both bandicoot species showed vivid pink photoluminescence over their entire pelts (Fig. 3). The ventral fur was a uniform pink, and the brindled flank fur shafts generally had a magenta photoluminescent base, a middle section that did not photoluminesce, and a yellowish photoluminescent tip. Other strands were wholly pink. The northern quoll and coppery brushtail possum also photoluminesced pink at 395–410 nm excitation, whereas the Lumholtz’s tree-kangaroo and pale field rat photoluminesced only blue (Fig. 3). The brushtail possum fur also photoluminesced purplish blue-grey, but only at 310–365 nm excitation. The platypus photoluminesced dully compared to the other species, but displayed colours of green/blue, and mild pink when clipped fur was viewed close up.

### Luminophore extraction

Shaved fur samples were soaked in a 20% TFA/water solution and heated at 95.0°C to extract the luminophores, and photoluminescence was evident in the extract (S1 Fig). The fur of all species still exhibited varying amounts of photoluminescence after extraction. After separating the fur and extract layers by centrifugation, a layer of coloured liquid was visible above the fur in white light for all species, and these layers photoluminesced under 365–410 nm light. When examined under 310 nm light, the fur extracts of the northern brown bandicoot and the Lumholtz’s tree-kangaroo photoluminesced pale purple. This purple photoluminescence was evident in the tree-kangaroo fur extract up to 410 nm excitation, but was eclipsed by hot pinkish orange photoluminescence using ≥  365 nm wavelength light in the bandicoot fur extract.

### RP-HPLC

**Fig. 4:**
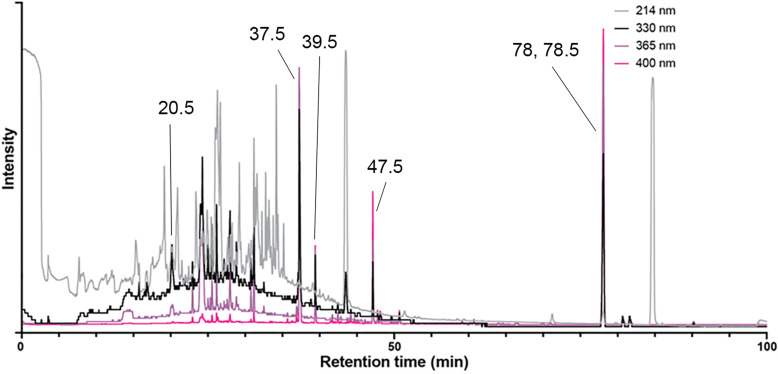
Example of a RP-HPLC chromatogram of northern long-nosed bandicoot (*Perameles pallescens*) fur extract. Monitored at 214, 330, 365 and 400 nm. Intensity levels for each wavelength optimized for clarity. Retention times (min) are highlighted for collected fractions containing easily visible peaks that showed photoluminescence. Left column: under white light; right column: under 310–410 nm ultraviolet-violet excitation (multiple torches). The tubes on the far right in each of (g) to (n) are non-photoluminescent control RP-HPLC fractions. (a) and (b) Northern long-nosed bandicoot (*Perameles pallescens*) (with pelt sample). Respective HPLC fraction retention times (L-R): 20.5, 23, 37.5, 39.5, 47.5, 78, 78.5 min. (c) and (d) Northern brown bandicoot (*Isoodon macrourus*) (with pelt sample). Respective HPLC fraction retention times (L-R): 20.5, 23, 37.5, 38, 40, 48, 78 min. (e) and (f) Northern quoll (*Dasyurus hallucatus*). Respective HPLC fraction retention times (L-R): 21, 23.5, 38, 48, 78.5 min. (g) and (h) Lumholtz’s tree-kangaroo (*Dendrolagus lumholtzi*). Respective HPLC fraction retention times (L-R): 23, 24.5. 25, 25.5, 26, 32, 34, 36.5, 38, 38.5, 76.5 min and control. (i) and (j) Coppery brushtail possum (*Trichosurus johnstonii*). Respective HPLC fraction retention times (L-R): 22.5, 23.5, 37, 38, 38.5, 39, 47, 77 min and control. (k) and (l) Pale field rat (*Rattus tunneyi*). Respective HPLC fraction retention times (L-R): 3, 3.5, 4, 20.5, 29.5, 47.5, 76 min and control. (m) and (n) Platypus (*Ornithorhynchus anatinus*). Respective HPLC fraction retention times (L-R): 2, 21.5, 24.5, 40, 44, 51, 55, 81.5 min and control.

**Fig. 5:**
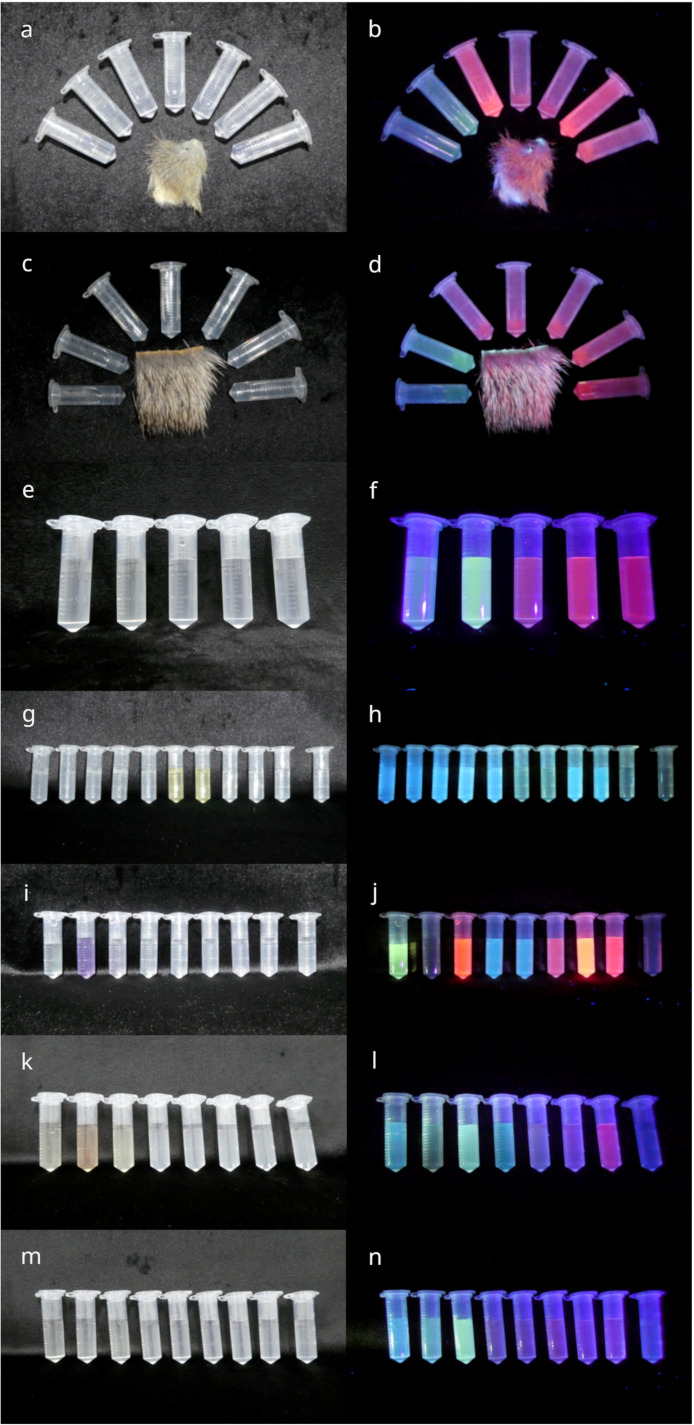
Selected RP-HPLC fractions in order of retention time.

Extracts from fur samples were run on RP-HPLC to separate and purify the luminophores. The retention times of the main peaks in the chromatograms (Fig. 4 and S2–7 Figs) at four different absorbance wavelengths for each species are listed in S2 Table. Some peaks were common across several species, showing similar retention times. For example, a peak with a retention time of ~  43 minutes at 214 nm absorbance was present in the chromatograms of the northern long-nosed bandicoot, the northern quoll, the coppery brushtail possum, the Lumholtz’s tree kangaroo and the pale field rat. Peaks at 37, 39, 47 and 78 minutes at 400 nm absorbance were present in both bandicoots and the possum, but only the ~  47- and 78-minute peaks were present in the quoll. Numerous luminophores were present in each sample, as evident from RP-HPLC fractionation and presence of photoluminescent compounds distributed across fractionation well-plates (S8 Fig). Fractions containing any colour under white light or photoluminescence under ultraviolet-violet wavelengths were removed from the plates and stored individually in micro-centrifuge tubes at 4°C. Figure 5 shows selected isolated fractions for each species under white light and 310–410 nm ultraviolet-violet light, respectively. The predominant photoluminescence was mostly pink or orange-pink, which was optimally excited at 395–410 nm. The Lumholtz’s tree-kangaroo fractions photoluminesced mostly blue or lavender blue (Fig. 5h), but two fractions that were yellow under white light weakly photoluminesced green (Fig. 5g and h). No other species yielded fractions that appeared blue in the well plates. However, once transferred to individual tubes, some greenish fractions appeared blueish (Fig. 5j, l and n, S3 Table). The coppery brushtail possum yielded non-photoluminescent fractions that were distinctly purple under white light (Fig 5i and j).

### LC/ESI-MS

The isolated fractions from each species were analysed by LC/ESI-MS to obtain mass and purity data for each fraction. The photoluminescent characteristics and molecular masses for each fraction are listed in S3 Table. Based on a previous report of protoporphyrin IX present in European hedgehog spine samples [35], a protoporphyrin IX standard was also analysed by LC/ESI-MS (S9 Fig). Protoporphyrin IX eluted with a retention time of 66.8 minutes and had a [*M* + H]^ +^ of *m/z* 563.3271 (theoretical exact mass 562.2580 Da).

A fraction corresponding to a chromatogram peak with a retention time of ~  38 min at 400 nm absorption was present for both species of bandicoot and the coppery brushtail possum. These fractions photoluminesced orange-pink and had monoisotopic masses of 830.2542 Da (northern long-nosed bandicoot), 830.3344 Da (northern brown bandicoot) and 830.3026 Da (coppery brushtail possum), which is consistent with the exact mass of uroporphyrin (830.2283 Da). A fraction corresponding to a chromatogram peak with a retention time of 30 minutes at 370 nm absorption was present for the northern long-nosed bandicoot and the northern quoll. These fractions photoluminesced pink and had monoisotopic masses of 654.3096 Da and 654.3324 Da respectively, which are consistent with the exact mass of coproporphyrin (654.2690 Da). A fraction corresponding to a chromatogram peak with a retention time of ~  41 minutes at 365 nm absorption was also present for the northern long-nosed bandicoot. This fraction photoluminesced pink and had a monoisotopic mass of 786.2855 Da, which is consistent with the exact mass of heptacarboxylporphyrin I (**12**) (786.2385 Da).

All species had a late-eluting peak in the 370 nm absorption chromatogram at ~  67 minutes that had a monoisotopic mass consistent with the exact mass of protoporphyrin (562.2580 Da). A comparison of the LC/ESI-MS chromatograms of the protoporphyrin IX standard and the fraction for the northern brown bandicoot predicted to contain protoporphyrin showed the monoisotopic mass (562.3062 Da) and retention time (66.6 minutes) of the sample fraction was consistent with the protoporphyrin IX standard (S9 Fig).

The pale field rat yielded two fractions at retention times of 4 and 21 minutes that luminesced strong canary yellow and lemon yellow and contained [*M* + H]^ +^ peaks of *m/z* 190.0262 and *m/z* 251.0517, respectively. These molecular ions are consistent with the exact masses of kynurenic acid (**3**) (189.0426 Da) and *N*-acetylkynurenine (250.0954 Da).

Seven molecules had masses unlikely to belong to the tryptophan derivative or porphyrin luminophore classes, and the remaining approximately 57 molecules isolated from fur remain unknown. Of the seven molecules unlikely to be tryptophan derivatives or porphyrins, examples include a fraction corresponding to a chromatogram peak with a retention time of ~  38 minutes at all absorption wavelengths present in the coppery brushtail possum and the Lumholtz’s tree-kangaroo. In the coppery brushtail possum, this fraction photoluminesced pale green and had a monoisotopic mass of 1342.7819 Da, and in the Lumholtz’s tree-kangaroo this fraction photoluminesced light blue at 365 nm or light yellow at 395–410 nm excitation and had a monoisotopic mass of 1376.7400 Da, not close to any of the known luminophores in fur. As an aside, one coppery brushtail possum fraction that did not photoluminesce, but displayed a distinct purple colour in white light, contained a molecule with a monoisotopic mass of 262.0422 Da, consistent with the exact mass of Indigo ((2E)-2-(3-Oxo-1,3-dihydro-2H-indol-2-ylidene)-1,2-dihydro-3H-indol-3-one; 262.0742 Da).

## Discussion

We observed photoluminescence in the fur of seven Australian Wet Tropics mammal species, including northern long-nosed and northern brown bandicoots, northern quoll, coppery brushtail possum, Lumholtz’s tree-kangaroo, pale field rat, and platypus. Analysis of fur extracts by RP-HPLC and LC/ESI-MS showed the presence of molecules consistent with uroporphyrin, heptacarboxylporphyrin, coproporphyrin and protoporphyrin, and aligns with findings in other species [16,35]. The strongly pink-photoluminescent species showed the presence of protoporphyrin and at least one other known porphyrin molecule, while the only known porphyrin in the species that did not photoluminesce strongly pink was protoporphyrin. These results support the hypothesis that the bright pink fur photoluminescence in bandicoots and at least two other species of Australian marsupial is caused by the presence of multiple porphyrin compounds in the pelage of each species.

The presence of porphyrins in the fur of marsupial and placental mammals from different continents [16,35,36] suggests the molecules are not species-specific and their combination and concentration can lead to varying colouration and intensity of photoluminescence in each species. Protoporphyrin was present in all species in this study, but the intensity differed and was lower in species that did not have a visible pink photoluminescence in the fur. It is possible that protoporphyrin is a ubiquitous compound in fur that varies in concentration in different species. Small amounts of protoporphyrin IX (**9**), as a precursor to haem, are ubiquitous in living cells [41]. Coproporphyrin and uroporphyrin are readily excreted in urine [42,43], but because of its insolubility in water, excess protoporphyrin IX (**9**) is not excreted through urine but in faeces [43] and through the liver, which can be problematic [41]. To further prevent toxic levels building up in the body, the pelage has been suggested as an unexplored additional metabolic pathway for the excretion of various porphyrins [36].

The metabolic pathways resulting in the accumulation of luminophores in fur are little understood. Tryptophan must be gained from an animal’s diet [44]; however, porphyrins can be obtained from diet [45,46] but are also synthesised internally [43,47]. Hamchand et al. (2021) proposed that the porphyrins in European hedgehogs originated from commensal microbes because spines of two red-photoluminescent European hedgehogs possessed different bacterial microbiomes to the quills of two non-photoluminescent American porcupines (*Erethizon dorsatum*) [35]. However, red photoluminescence has not been found on the skin of European hedgehogs [11,35]. Toussaint et al. (2023) found the distribution of red photoluminescence to indicate that the porphyrins are more likely incorporated into hedgehog spines during growth [36], as are tryptophan luminophores into the fur of other mammals [14,25].

We identified molecules consistent with uroporphyrin and protoporphyrin in an extract of coppery brushtail possum fur, whereas Nicholls and Rienits (1971) did not identify any porphyrins in the fur of the closely related common brushtail possum [25]. Common brushtail possum fur was recorded as having reddish photoluminescence by Bolliger (1944) [26], but not by Nicholls and Rienits (1971) [25]. However, porphyrins may have been missed because of the use of only water as a solvent in the extraction process, an excitation wavelength too short for the luminophores, or because of photobleaching [48]. The highly hydrophobic nature of protoporphyrins [41] also means they may have been missed in previous chromatography studies due to a much longer retention time than other porphyrin molecules.

Of approximately 70 molecular ions identified in the fur extractions, only four could be attributed to the porphyrins coproporphyrin, protoporphyrin, uroporphyrin and heptacarboxylporphyrin previously identified in mammalian pelage. Two molecules were consistent with the tryptophan metabolites kynurenic acid and *N*-acetylkynurenine previously found in laboratory rat fur [28,29]. Seven molecular ions were consistent with masses of molecules that may not belong to either of these two luminophore classes, although these remain unconfirmed. The remaining molecules ( ~ 57) potentially contributing to variously coloured photoluminescence in the majority of sample fractions from all species could not be identified. This conclusion is consistent with Nicholls and Rienits’ (1971) observation related to the confusing number of fluorescent components present in some samples [25], as well as the multiple potential luminophores in squirrel fur [37]. Identification of fur luminophores, their metabolic pathways, and potential for photoluminescence to be an indicator of condition, remain promising avenues of future research for mammals.

With the exception of porphyrins in hedgehog spines [11,35], tryptophan metabolites were long thought to be the only known luminophores in fur. Tryptophan metabolite luminophores have been previously identified from the fur of several mammal species, including a brushtail possum and a tree-kangaroo [14,25,29]. It was unexpected that in all the Wet Tropics species investigated here, molecular ions for only two tryptophan derivatives were identified in the photoluminescent fractions from the pale field rat only. Nicholls and Rienits (1971) reported the presence of 3-hydroxyanthranilic acid and kynurenine in the fur of the Goodfellow’s tree-kangaroo [25], but these compounds were not evident in our Lumholtz’s tree-kangaroo sample.

The luminophore colours evident in the photoluminescent RP-HPLC fractions were not necessarily evident in the fur. For the platypus, pink photoluminescence was only found on closer inspection of the remaining clipped fur after the isolation of pink luminophores. However, for the Lumholtz’s tree-kangaroo and the pale field rat, no pink fur photoluminescence could be detected by the human eye. Similarly, Hughes et al. (2022) found the fur extracts of otherwise non-photoluminescent squirrels to photoluminesce in solution [37]. Rebell et al. (1956) also noted a weak blueish photoluminescence in the fur extracts of several domestic species, even if there was no photoluminescence evident when viewed as whole animals [27], but the cause of this general blueish photoluminescence was not resolved. Conversely, for the brightly blue-photoluminescent pale field rat, the blue colour was not evident in the RP-HPLC well-plate fractions. However, once some of the fractions of the pale field rat, the coppery brushtail possum and the platypus were transferred from the well plates into individual Eppendorf tubes, the yellowish or greenish fractions became blue-shifted, both to the human eye and to the camera. Further analysis of these fractions could be informative.

Differences between the appearance of luminophores extracted in solution compared to when present in the fur were observed. These discrepancies could be due to differences in microenvironment between the fur and the solvent. For example, kynurenine becomes more photoluminescent when it is bound to proteins, being barely photoluminescent in its free state [49]. For tryptophan residues within proteins, the microenvironment (hydration, softening of the keratin matrix, breakage of disulfide bonds, interactions with some enzymes and side-chain amino acids) can either quench or boost the intensity of photoluminescence [50,51]. The presence of other compounds, such as melanin, can also act to quench the photoluminescence in fur [18,29,52]. The isolation of luminophores not otherwise visible in the fur itself suggests that those luminophores have no optical function *in situ*.

## Conclusions

In this study, we examined the luminophore composition of photoluminescent fur from seven species of mammal from the Wet Tropics of Far North Queensland, Australia. Numerous luminophores were present in each fur sample. Some luminophores were common across different species, with protoporphyrin identified in all species tested. A luminophore consistent with uroporphyrin was also identified in both pink-photoluminescent species of bandicoot, heptacarboxylporphyrin in one bandicoot, and coproporphyrin in a bandicoot and the pink-photoluminescent northern quoll. We only isolated two luminophores with molecular ions consistent with known tryptophan metabolites that have been documented previously in the pelage of other mammals. However, our results provide evidence for the presence of several luminophores that remain to be identified or confirmed. For some species, the observed colour of the fur photoluminescence or fur extraction was not necessarily predictive of the individual luminophores present. This is the first study to indicate the extent of the mostly unidentified luminophore composition across species of marsupial, monotreme and placental mammal fur from one bioregion of Australia.

## Supporting information

S1 Table Amounts of fur, solvent and extract used for the RP-HPLC of each species. Shaved fur samples were soaked in a 20% TFA/water solution and heated at 95.0°C to extract the luminophores.
(DOCX)

S2 Table Approximate retention times (min) of the highest intensity RP-HPLC peaks from the RP-HPLC chromatograms at various absorbance wavelengths for each species. (DOCX)

S3 Table Molecular ions [*M* + H] ^+^ and colours of selected fur extract RP-HPLC fractions. ‘Potential compounds’ suggests compounds that are consistent with the mass of the molecule extracted from fur.
(DOCX)

S1 Fig Example of fur luminophore extraction in 20% TFA, after heating for one hour at 95.0°C. Coppery brushtail possum fur extraction (*Trichosurus johnstonii*) under (a) white light and (b) 395–410 nm ultraviolet-violet light. (TIF)

S2 Fig RP-HPLC chromatogram of fur extract monitored at 214, 330, 365 and 400 nm. Intensity levels for each wavelength optimized for clarity. Northern brown bandicoot (*Isoodon macrourus*).
(TIF)

S3 Fig RP-HPLC chromatogram of fur extract monitored at 214, 330, 365 and 400 nm. Intensity levels for each wavelength optimized for clarity. Northern quoll (*Dasyurus hallucatus*).
(TIF)

S4 Fig RP-HPLC chromatogram of fur extract monitored at 214, 330, 365 and 400 nm. Intensity levels for each wavelength optimized for clarity. Coppery brushtail possum (*Trichosurus johnstonii*).
(TIF)

S5 Fig RP-HPLC chromatogram of fur extract monitored at 214, 330, 365 and 400 nm. Intensity levels for each wavelength optimized for clarity. Lumholtz’s tree-kangaroo (*Dendrolagus lumholtzi*).
(TIF)

S6 Fig RP-HPLC chromatogram of fur extract monitored at 214, 330, 365 and 400 nm. Intensity levels for each wavelength optimized for clarity. Pale field rat (*Rattus tunneyi*).
(TIF)

S7 Fig RP-HPLC chromatogram of fur extract monitored at 214, 330, 365 and 400 nm. Intensity levels for each wavelength optimized for clarity. Platypus (*Ornithorhynchus anatinus*).
(TIF)

S8 Fig RP-HPLC fractions of a sample from a northern long-nosed bandicoot (*Perameles pallescens*). Observed under a 395–410 nm ultraviolet-violet torch showing the presence of photoluminescent compounds in individual wells. (JPG)

S9 Fig LC/ESI-MS total ion current chromatograms (positive ion mode), 370 nm UV chromatograms and mass spectra. (a) Northern brown bandicoot fraction plate P2E12. (b) Protoporphyrin IX standard.
(JPG)
