# Supplementary material for: Luminophores in the fur of seven Australian Wet Tropics mammals
Source: PLoS One. 2025 Apr 30;20(4):e0320432. doi: 10.1371/journal.pone.0320432 (PMC12043139; doi:10.1371/journal.pone.0320432)
Supplement: S1 Table — Shaved fur samples were soaked in a 20% TFA/water solution and heated at 95.0°C to extract the luminophores. (DOCX) [file pone.0320432.s001.docx]

**S1 Table.** Amounts of fur, solvent and extract used for the RP-HPLC of each species. Shaved fur samples were soaked in a 20% TFA/water solution and heated at 95.0°C to extract the luminophores.

| Species | Fur mass  mg | Solvent volume  mL | Extract volume  mL |
| --- | --- | --- | --- |
| Northern long-nosed bandicoot (*Perameles pallescens*) | 258.38 | 8 | 5 |
| Northern brown bandicoot (*Isoodon macrourus*) | 793.18 | 12 | 6 |
| Northern quoll (*Dasyurus hallucatus*) | 758.37 | 7 | 4.5 |
| Coppery brushtail possum (*Trichosurus johnstonii*) | 387.02 | 7 | 5 |
| Lumholtz’s tree-kangaroo (*Dendrolagus lumholtzi*) | 1166.94 | 10 | 6 |
| Pale field rat (*Rattus tunneyi*) | 400.85 | 7 | 5 |
| Platypus (*Ornithorhynchus anatinus*) | 520.05 | 7 | 4.5 |
