# Supplementary material for: Luminophores in the fur of seven Australian Wet Tropics mammals
Source: PLoS One. 2025 Apr 30;20(4):e0320432. doi: 10.1371/journal.pone.0320432 (PMC12043139; doi:10.1371/journal.pone.0320432)
Supplement: S2 Table — (DOCX) [file pone.0320432.s002.docx]

**S2 Table.** Approximate retention times (min) of the highest intensity RP-HPLC peaks from the RP-HPLC chromatograms at various absorbance wavelengths for each species.

| **Species / absorbance** | **214 nm** | **330 nm** | **365 nm** | **400 nm** |
| --- | --- | --- | --- | --- |
| Northern long-nosed bandicoot (*Perameles pallescens*) | 43, 85 | – | – | 37, 39, 47, 78 |
| Northern brown bandicoot (*Isoodon macrourus*) | 51 | – | – | 37, 39, 48, 78 |
| Northern quoll (*Dasyurus hallucatus*) | 44, 85 | – | – | 48, 78 |
| Coppery brushtail possum (*Trichosurus johnstonii*) | 43, 51 | 2, 12, 19, 23, 25, 27, 28, 37 | 2, 12, 19, 22, 25, 27, 28, 30, 37 | 37, 39, 46, 77 |
| Lumholtz’s tree-kangaroo (*Dendrolagus lumholtzi*) | 43, 51 | 20, 25 | 20 | 31, 34, 36 |
| Pale field rat (*Rattus tunneyi*) | 3, 13, 20, 29, 31, 42 | 3, 20 | 3, 20 | 3, 20 |
| Platypus (*Ornithorhynchus anatinus*) | 46 | 2 | 2 | 2 |
