# Supplementary material for: Luminophores in the fur of seven Australian Wet Tropics mammals
Source: PLoS One. 2025 Apr 30;20(4):e0320432. doi: 10.1371/journal.pone.0320432 (PMC12043139; doi:10.1371/journal.pone.0320432)
Supplement: S3 Table — ‘Potential compounds’ suggests compounds that are consistent with the mass of the molecule extracted from fur. (DOCX) [file pone.0320432.s003.docx]

**S3 Table.** Molecular ions [*M*+H]^+^ and colours of selected fur extract RP-HPLC fractions. ‘Potential compounds’ suggests compounds that are consistent with the mass of the molecule extracted from fur.

| **Species** | **RP-HPLC**  **fraction** | **Colour**  (colourless in white light unless stated) | **Molecular ions** **observed (m/z)** | **Potential**  **compounds** |
| --- | --- | --- | --- | --- |
| Northern long-nosed bandicoot  (*Perameles pallescens*) | P1 D5 | Photoluminescent mild yellow. | 501.2937  295.1647  381.1709  408.1499  533.2503  516.2367  432.2104  603.2643  630.2874 | ?  ?  ?  ?  ?  ?  ?  ?  ? |
|  | P1 D10 | Photoluminescent mild yellow. | 246.1843  377.1408  352.1740  523.2879  761.4003 | ?  Riboflavin?  ?  ?  ? |
|  | P1 E5 | Non-photoluminescent pink visible in white light. | 606.3652  386.1760  1016.4341  473.1981  530.2732  572.2774  629.3230  743.3622  686.3218 | ?  ?  ?  ?  ?  ?  ?  ?  ? |
|  | P1 G3 | Photoluminescent hot pink (pink visible in white light). | 347.2253  831.2615  274.2733 | ?  Uroporphyrin  ? |
|  | P1 G7 | Photoluminescent bright pink. | 787.2928  274.2935 | Heptacarboxylporphyrin  ? |
|  | P1 H11 | Photoluminescent bright pink. | 453.3703  274.3042  655.3169 | ?  ?  Coproporphyrin |
|  | P2 F1 | Photoluminescent hot pink (pink visible in white light). | 274.2903  563.3058 | ?  Protoporphyrin |
| Northern brown bandicoot  (*Isoodon macrourus*) | P1 D5 | Photoluminescent pastel yellow. | 246.0678  508.3100 | ?  ? |
|  | P1 D10 | Photoluminescent pastel yellow. | 246.1228  329.0957  370.1583 | ?  ?  ? |
|  | P1 G3 | Photoluminescent hot orangey pink. | 327.1747  347.1721  831.3417  715.5227 | ?  ?  Uroporphyrin  ? |
|  | P1 G8 | Photoluminescent hot orangey pink. | 327.1825  367.1854  390.2439  787.3524 | ?  ?  ?  ? |
|  | P1 H12 | Photoluminescent hot orangey pink. | 243.1544  261.1779  266.1638  283.1519  323.1380  365.1554  623.4126 | ?  ?  ?  ?  ?  ?  ? |
|  | P2 C1 | Photoluminescent pink. | 283.1330  332.3017  346.1987  326.3395  5083.1102 | ?  ?  ?  ?  ? |
|  | P2 E12 | Photoluminescent hot orangey pink. | 563.3135  663.5086  704.6407  5083.2729 | Protoporphyrin  ?  ?  Peptide? |
| Northern quoll  (*Dasyurus hallucatus*) | P1 A4 | Photoluminescent yellow (dark yellow visible in white light). | 614.7210 | ? |
|  | P1 D6 | Photoluminescent yellow. | Inconclusive | ? |
|  | P1 D11 | Photoluminescent very bright yellow (dark yellow visible in white light). | 508.2770  595.3334 | ?  ? |
|  | P1 G4 | Photoluminescent pink. | 2683.6670 | Peptide? |
|  | P1 H12 | Photoluminescent bright pink. | 655.3397 | Coproporphyrin |
|  | P2 F1 | Photoluminescent bright pink. | 563.3103  5082.6775  5067.2158 | Protoporphyrin  Peptide  Peptide |
| Coppery brushtail possum  (*Trichosurus johnstonii*) | P1 D9 | Photoluminescent bright yellow [appeared more green when transferred to tube] (mild dark yellow visible in white light). | 329.1003  909.5227  370.1376  215.0978 | ?  ?  ?  ? |
|  | P1 D10 | Non-photoluminescent violet visible in white light. | 647.3729  263.0495  215.0978  299.0722 (-)  413.1016 (-)  599.2337 (-) | ?  Indigo  ?  ?  ?  ? |
|  | P1 D11 | Non-photoluminescent strong violet visible in white light. | 215.0676  409.1833  299.0722 (-)  413.1016 (-)  599.2337 (-) | ?  ?  ?  ?  ? |
|  | P1 G2 | Photoluminescent bright hot orange-pink (dusky pink visible in white light). | 2481.1596  1286.6669  831.3099  1117.5364 | Peptide?  ?  Uroporphyrin  ? |
|  | P1 G5 | Photoluminescent pale green [the green fractions G4 and G5 appeared blue when transferred to tubes]. | 1343.7892  1400.8996  1001.4962  408.2166 | ?  ?  ?  ? |
|  | P1 H10 | Photoluminescent bright hot orange-pink. | failed | ? |
|  | P2 E10 | Photoluminescent pink. | 563.3113 | Protoporphyrin |
| Lumholtz’s tree-kangaroo  (*Dendrolagus lumholtzi*) | P1 A2 | Photoluminescent very slightly brownish. | 316.9976 | ? |
|  | P1 D10 | Photoluminescent slightly brownish. | 909.5470 | ? |
|  | P1 E2 | Photoluminescent pale blue in 395–410 nm light; pale lavender blue in 365 nm light. | 629.3307 | ? |
|  | P1 E3 | Photoluminescent pale orange in 395–410 nm light; pale lavender blue in 365 nm light. | 386.1335  443.1900  603.3502 | ?  ?  ? |
|  | P1 F4 | Photoluminescent dark yellow. | 544.2568 | ? |
|  | P1 F8 | Photoluminescent dark yellow (yellow visible in white light). | 791.4106  800.4366 | ?  ? |
|  | P1 G1 | Photoluminescent dark yellow. | 743.3970  301.0101  1283.5164 | ?  ?  ? |
|  | P1 G4 | Photoluminescent light yellow in 395–410 nm light; very light blue in 365 nm light. | 1377.7473 | ? |
|  | P2 E9 | Photoluminescent slight dusky pink. | 5067.1062  5083.0275  379.3242  563.6330 | Peptide?  Peptide?  ?  Protoporphyrin |
| Pale field rat  (*Rattus tunneyi*) | P1 A6 | Photoluminescent slight greenish pastel yellow [appeared pale greenish blueish white in tube] (dark yellow visible in white light). | 314.1432 | ? |
|  | P1 A7 | Photoluminescent dark brown with yellow (rusty orange/ reddish brown visible in white light). | 250.0655 | ? |
|  | P1 A8 | Photoluminescent strong canary yellow (dark yellow visible in white light). | 187.0501  190.0362  231.0492 | ?  Kynurenic acid?  ? |
|  | P1 D5 | Photoluminescent lemon yellow [appeared pale greenish in tube]. | 251.0517  292.0798 | N-acetylkynurenine?  ? |
|  | P1 E11 | Photoluminescent faint orange-pink. | 514.2827 | ? |
|  | P1 H11 | Photoluminescent faint pink. | 326.3345  332.2833 | ?  ? |
|  | P2 A4 | Photoluminescent faint pink. | 5083.9004 | ? |
|  | P2 E8 | Photoluminescent bright orange [appeared bright pink in tube.] | 563.3157 | Protoporphyrin |
| Platypus  (*Ornithorhynchus anatinus*) | P1 A4 | Photoluminescent very faint yellow [appeared cyan in tube] (mild dark yellow visible in white light). | 249.0177  316.9849 | ?  ? |
|  | P1 D7 | Photoluminescent yellow [appeared cyan in tube] (very faint dark yellow visible in white light). | 246.0642  221.1065  254.0855 | ?  ?  ? |
|  | P1 E1 | Photoluminescent yellow (very faint dark yellow visible in white light). | 329.1053  370.1384 | ?  ? |
|  | P1 G8 | Photoluminescent faint pink (very faint dark yellow visible in white light). | 277.0861 | ? |
|  | P1 H4 | Photoluminescent faint pink. | – | ? |
|  | P2 A6 | Photoluminescent faint pink. | 233.1318  247.1357 | ?  ? |
|  | P2 B2 | Photoluminescent faint pink. | 285.2151  432.1786 | ?  ? |
|  | P2 F7 | Photoluminescent pink. | 563.3314 | Protoporphyrin |
