# Supplementary figures and images for: Luminophores in the fur of seven Australian Wet Tropics mammals
Source: PLoS One. 2025 Apr 30;20(4):e0320432. doi: 10.1371/journal.pone.0320432 (PMC12043139; doi:10.1371/journal.pone.0320432)

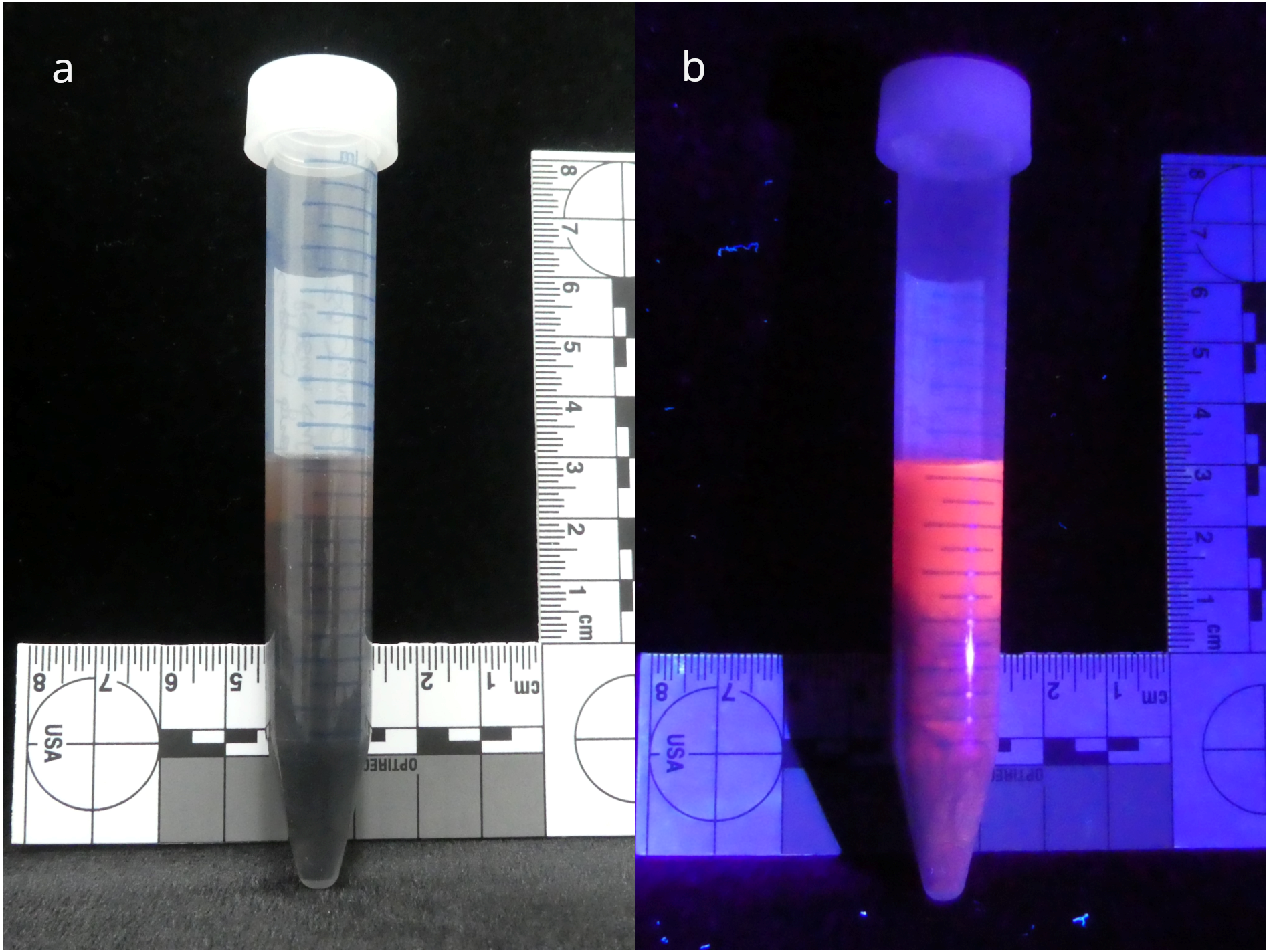

Supplement: S1 Fig — (TIF) [file pone.0320432.s004.tif]

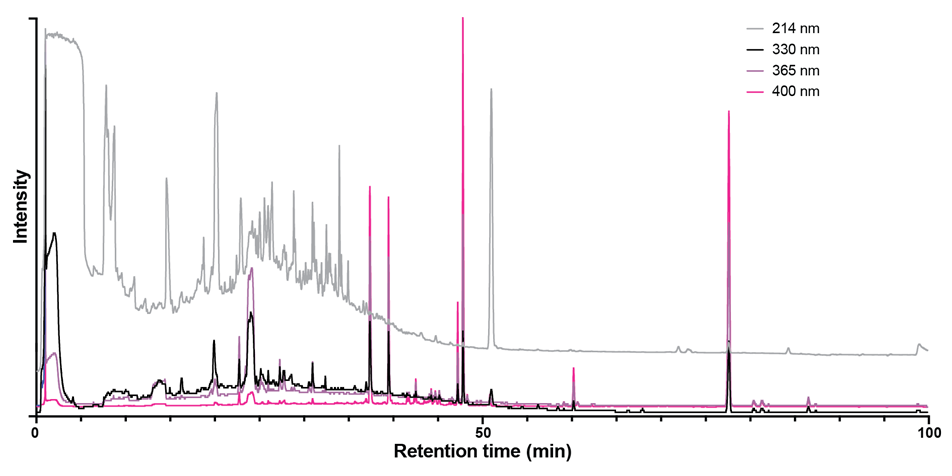

Supplement: S2 Fig — Northern brown bandicoot (Isoodon macrourus). (TIF) [file pone.0320432.s005.tif]

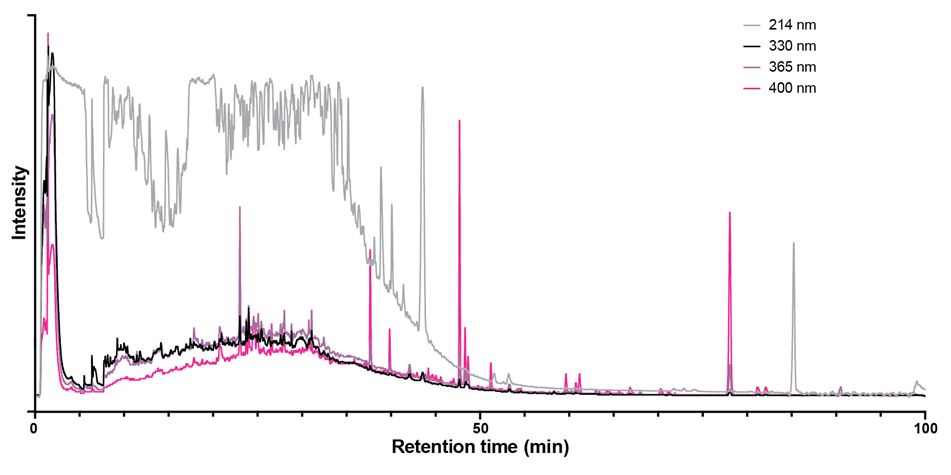

Supplement: S3 Fig — Northern quoll (Dasyurus hallucatus). (TIF) [file pone.0320432.s006.tif]

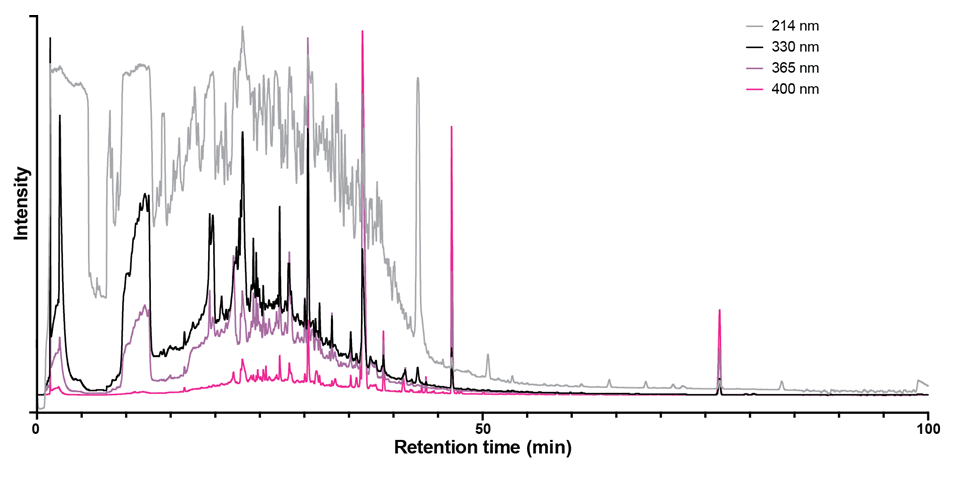

Supplement: S4 Fig — Coppery brushtail possum (Trichosurus johnstonii). (TIF) [file pone.0320432.s007.tif]

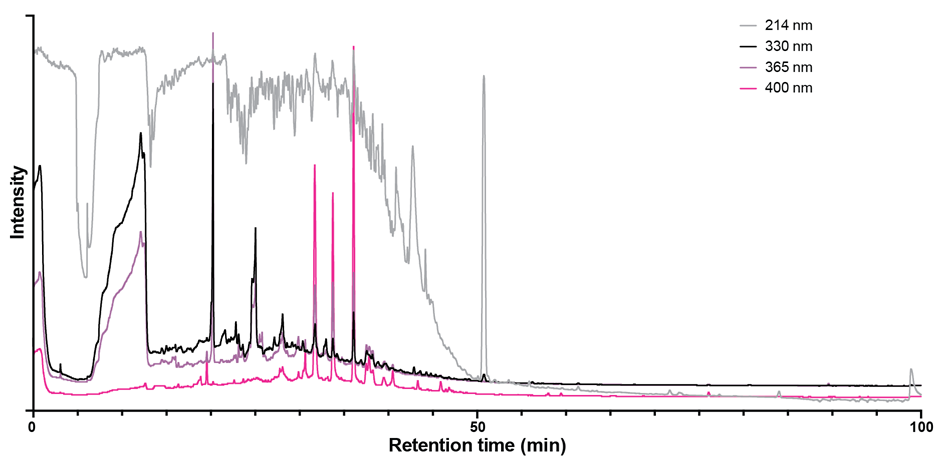

Supplement: S5 Fig — Lumholtz’s tree-kangaroo (Dendrolagus lumholtzi). (TIF) [file pone.0320432.s008.tif]

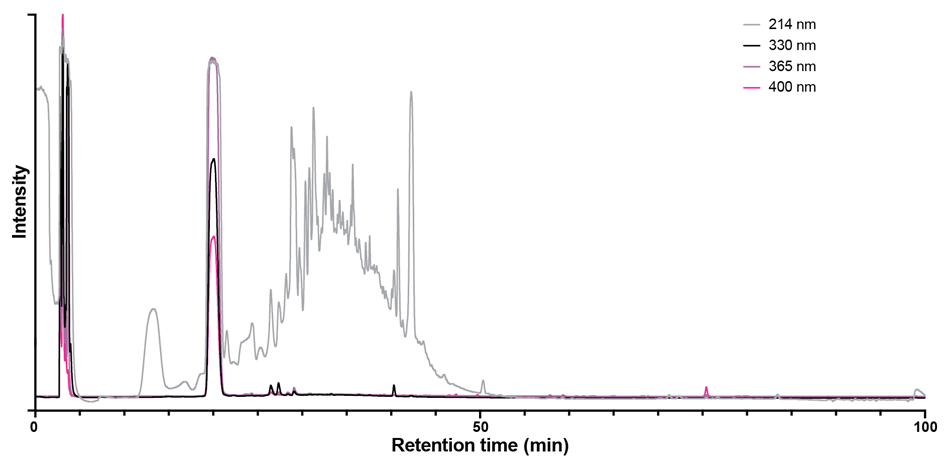

Supplement: S6 Fig — Pale field rat (Rattus tunneyi). (TIF) [file pone.0320432.s009.tif]

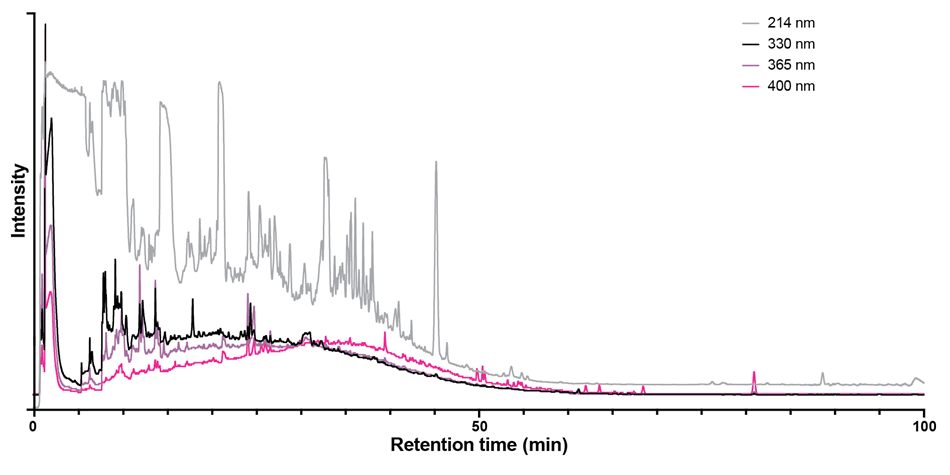

Supplement: S7 Fig — Platypus (Ornithorhynchus anatinus). (TIF) [file pone.0320432.s010.tif]

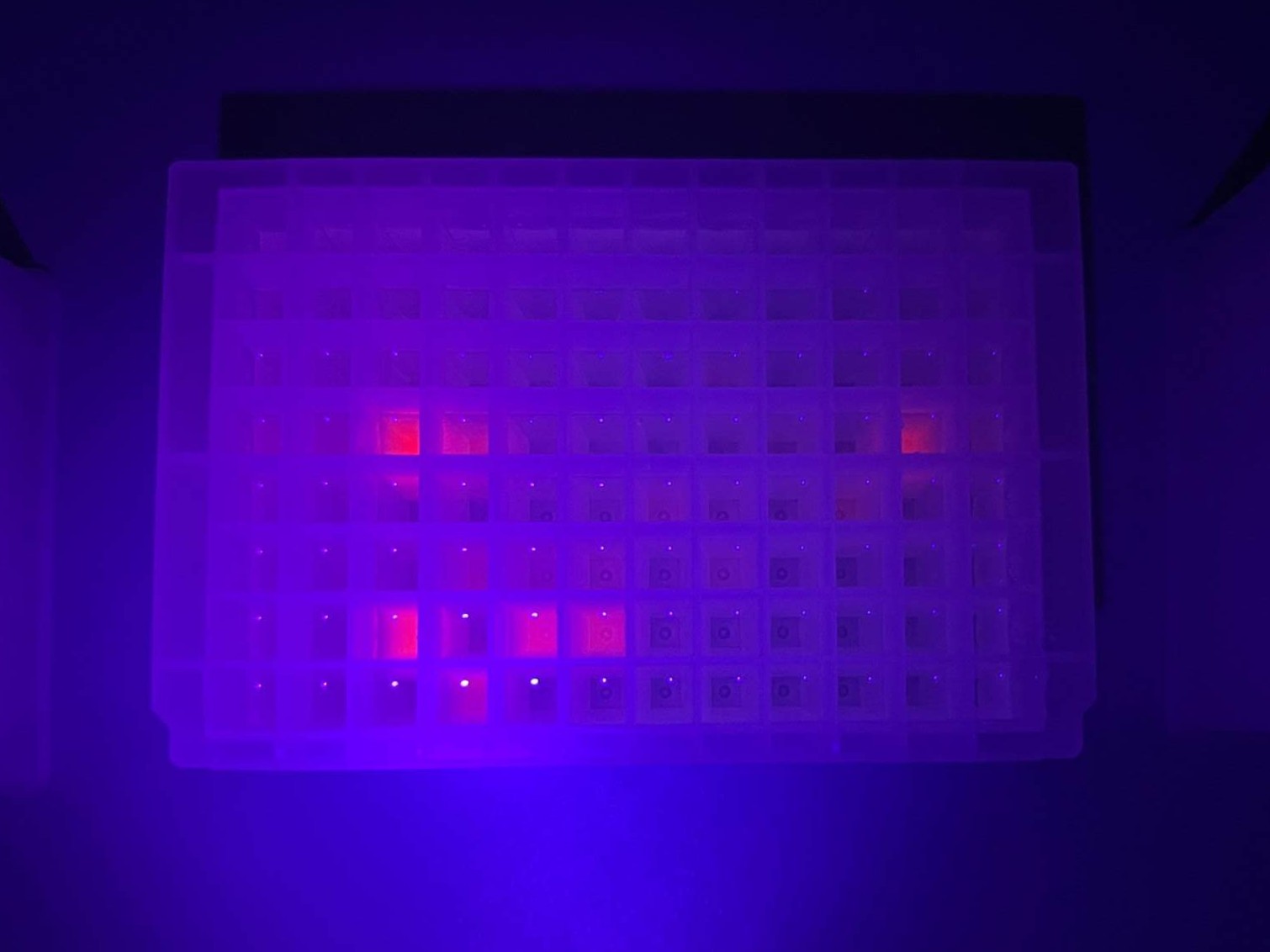

Supplement: S8 Fig — (JPG) [file pone.0320432.s011.jpg]

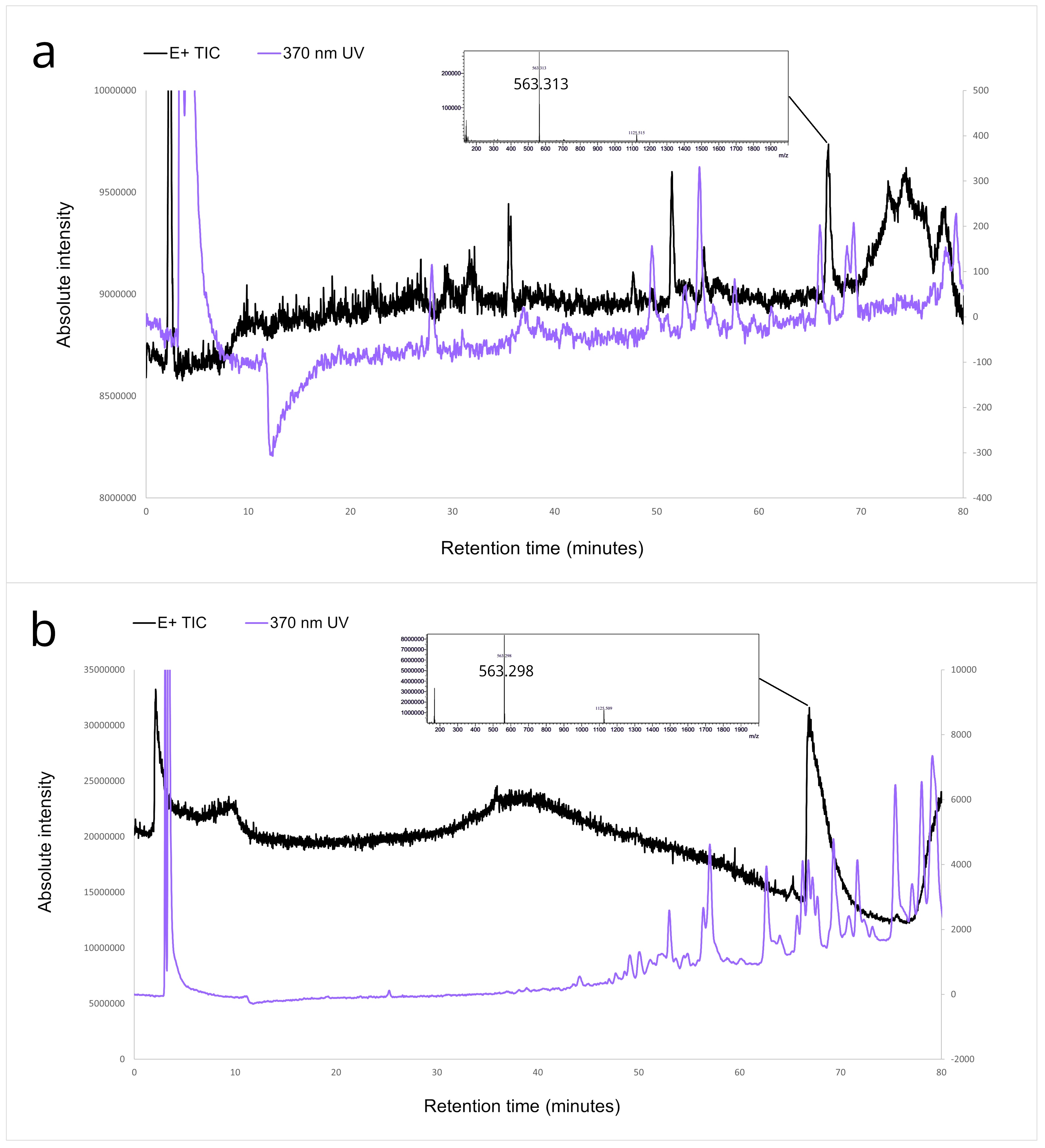

Supplement: S9 Fig — (b) Protoporphyrin IX standard. (JPG) [file pone.0320432.s012.jpg]
